# Retraction: Microwave-assisted synthetic method of novel Bi_2_O_3_ nanostructure and its application as a high-performance nano-catalyst in preparing benzylidene barbituric acid derivatives

**DOI:** 10.3389/fchem.2026.1959085

**Published:** 2026-08-14

**Authors:** Frontiers Editorial Office

The journal retracts the 07 October 2022 article cited above.

Following publication, concerns were raised about the integrity of the images in the published figures. The authors failed to provide a satisfactory explanation during the investigation, which was conducted in accordance with Frontiers' policies.

This retraction was approved by the Chief Executive Editor of Frontiers. The authors do not agree to this retraction.

Frontiers would like to thank the users on PubPeer for bringing the published article to our attention.

